# Development of integration indexes to determine the extent of family planning and child immunization services integration in health facilities in urban areas of Nigeria

**DOI:** 10.1186/s12978-021-01105-y

**Published:** 2021-02-23

**Authors:** Kate L. Sheahan, Jennifer Orgill-Meyer, Ilene S. Speizer, Siân Curtis, John Paul, Morris Weinberger, Antonia V. Bennett

**Affiliations:** 1 The Durham Center of Innovation to Accelerate Discovery and Practice Transformation (ADAPT), (CIN 13-410) at the Durham VA Health Care System, Durham, NC USA; 2 Franklin and Marshall College, Lancaster, Pennsylvania USA; 3 University of North Carolina at Chapel Hill, Chapel Hill, NC USA

**Keywords:** Family planning, Immunization, Integration, Measurement, Nigeria

## Abstract

**Background:**

Integrating family planning into child immunization services may address unmet need for contraception by offering family planning information and services to postpartum women during routine child immunization visits. However, policies and programs promoting integration are often based on insubstantial or conflicting evidence about its effects on service delivery and health outcomes. While integration models vary, many studies measure integration as binary (a facility is integrated or not) rather than a multidimensional and varying continuum. It is thus challenging to ascertain the determinants and effects of integrated service delivery. This study creates Facility and Provider Integration Indexes, which measure capacity to support integrated family planning and child immunization services and applies them to analyze the extent of integration across 400 health facilities.

**Methods:**

This study utilizes cross-sectional health facility (N = 400; 58% hospitals, 42% primary healthcare centers) and healthcare provider (N = 1479) survey data that were collected in six urban areas of Nigeria for the impact evaluation of the Nigerian Urban Reproductive Health Initiative. Principal Component Analysis was used to develop Provider and Facility Integration Indexes that estimate the extent of integration in these health facilities. The Provider Integration Index measures provider skills and practices that support integrated service delivery while the Facility Integration Index measures facility norms that support integrated service delivery. Index scores range from zero (low) to ten (high).

**Results:**

Mean Provider Integration Index score is 5.42 (SD 3.10), and mean Facility Integration Index score is 6.22 (SD 2.72). Twenty-three percent of facilities were classified as having low Provider Integration scores, 32% as medium, and 45% as high. Fourteen percent of facilities were classified as having low Facility Integration scores, 38% as medium, and 48% as high.

**Conclusion:**

Many facilities in our sample have achieved high levels of integration, while many others have not. Results suggest that using more nuanced measures of integration may (a) more accurately reflect true variation in integration within and across health facilities, (b) enable more precise measurement of the determinants or effects of integration, and (c) provide more tailored, actionable information about how best to improve integration. Overall, results reinforce the importance of utilizing more nuanced measures of facility-level integration.

## Plain English summary

Inadequate spacing between pregnancies can lead to adverse health outcomes among women and babies. The WHO recommends an interval of 24 months between pregnancies. Access to postpartum contraception is critical, yet many women wishing to space their pregnancies do not use it. Integrating family planning services into child immunization services may increase access to postpartum contraception. While previous research shows that integration is acceptable to women, evidence about its effects on service delivery and health outcomes is scarce and inconsistent. This is due, in part, to challenges surrounding the measurement of integration. One such challenge is that integration within health facilities is often measured as binary (i.e., a facility is designated as integrated or not), though numerous factors influence the extent of integration within facilities over time. Without capturing variation in integration, analyzing the effects of integration is difficult. This study addresses that challenge by developing Provider (i.e., nurse/midwife) and Facility Integration Indexes, which measure capacity to support integrated family planning and child immunization services. Utilizing cross-sectional data collected for the Nigerian Urban Reproductive Health Initiative, we apply the indexes to describe the extent of integration across 400 facilities: 23% of facilities have low Provider Integration scores, 32% have medium, and 45% have high. Fourteen percent of facilities have low Facility Integration scores, 38% have medium, and 48% have high. These results suggest that nuanced measures of integration, like those described in this paper, may enable more accurate evaluation of integration’s effects, and provide more specific information about whether and how to support integration.

## Introduction

Nigeria has among the highest maternal and infant mortality rates in the world. In 2017, Nigeria had a maternal mortality ratio (MMR) of 917/100,000 live births, which was the fifth highest in the world [1]. At the same time, Nigeria’s infant mortality rate (IMR) was 67/1000 live births [2]. Nigeria signed on, and recently reconfirmed its resolve, to achieve Sustainable Development Goal 3 to reduce MMR to below 70/100,000 live births and to end preventable deaths of newborns and children under age 5 years by 2030 [3].

Family planning use has the potential to drastically reduce newborn and maternal deaths globally [4]. Yet, unmet need for contraception remains high among Nigerian women; 24.8% of women of reproductive age (15–49 years) want to stop or delay childbearing but are not using contraception [5]. The Federal Government of Nigeria set a target to increase the modern contraceptive prevalence rate from 10% in 2012 to 27% by 2020 [6]. By 2018, the modern contraceptive prevalence rate among all women had increased to 13.8%, though this remains well below the target [5]. Thus, increasing access to and utilization of family planning methods remains an urgent priority in Nigeria.

### Contraception among postpartum women: critical to maternal and infant health

Postpartum contraceptive use is particularly critical to the health of women and babies. Interpregnancy intervals of fewer  than 18 months are associated with increased risk of infant and maternal morbidity and mortality [7]. Further, under-five mortality is significantly higher among children born fewer than 24-months after the preceding birth. One in four children in Nigeria are born less than 2 years after a sibling. The under-five mortality rate is 183/1000 among these children; this declines to 93/1000 among children born 3 years after the previous birth [2]. The World Health Organization (WHO) recommends an interval of at least 24 months after a live birth prior to attempting the next pregnancy [8]. In Nigeria, 65% of women who have given birth within the last 23 months may have an unmet need for contraception [9]. Meeting this postpartum need for contraception would contribute substantially to contraceptive prevalence, which would in turn decrease MMR and IMR.

### Integration of family planning and child immunization services

Integrating family planning into routine child immunization services has the potential to address unmet need for contraception among postpartum women by leveraging repeated immunization consultations to offer family planning information and services. Although modern contraceptive use in the postpartum period may be low in many contexts, immunization coverage is generally high. Immunization services may thus provide a solid platform for the integration of family planning services [10]. In 2019, 85% of infants globally received the recommended three doses of the diphtheria-tetanus-pertussis (DPT-3) vaccine, although coverage in Nigeria is lower at around 50% [11, 12]. While integration models vary in purpose and design, two primary models are commonly implemented [13, 14]. The first, combined service provision, entails offering both family planning and immunization services on the same day at the same location. The second, single service provision plus referral, entails offering either family planning or immunization services along with education, screening, and/or referral for the other service at a different place or time. Previous research has shown that integration can be feasible and acceptable to providers and clients [15–18]. However, numerous factors and challenges influence the extent of integration attained within a facility, including health system characteristics, provider and client characteristics, staffing and space constraints, and the cultural context [19–21].

Within health facilities, family planning services may be integrated into child immunization services at the primary and secondary levels. The Nigerian Ministry of Health promotes the integration of family planning into immunization services as an important approach to increasing family planning availability and accessibility [22]. The Minimum Standards for Primary Healthcare (MSPH) in Nigeria identify both family planning and immunization services as minimum components of primary healthcare and require provision of these services at all public primary healthcare facilities while advocating that privately owned facilities align with these standards [23]. Both immunization and family planning services form part of a standard package of primary healthcare services commonly provided within hospitals.

### Evidence and measurement gaps

Policy and programming recommendations that promote integrating family planning and child immunization services are often based on insubstantial or conflicting evidence about its impact on health services delivery and patient outcomes [24–27]. Given the scarcity of evidence and the resource and planning implications of integration, it is critical to develop context specific evidence that captures the nature, extent, and effects of integration in order to inform policy and program design [13].

The complexity of implementing integrated service delivery combined with the varied integration models and definitions prompt questions related to how integration and its effects should be measured [28–30]. Many studies measure integration as a binary variable (a facility is integrated or not) rather than a multidimensional continuum that varies across time and place [17, 18, 28]. A few studies have developed more nuanced measures of integration and assessed associations between these levels and health and service delivery outcomes. For example, the Integra Initiative developed indexes that measure the extent and type of HIV and reproductive health integration, and Church [20] analyzed whether degree of HIV and reproductive health integration was associated with client demand for services and unmet need for family planning [20, 30]. Mackenzie (2018) identified that the level of maternal, newborn, and child health and family planning integration varies widely across facilities as well as across clinical areas [31]. Multi-dimensional, continuous measures of integration are valuable because they enable analyses related to the extent of integration within and across facilities and the effects of varying degrees of integration on service delivery and health outcomes. No studies, to our knowledge, have measured the extent of family planning and child immunization services integration [32, 33]. This study aims to address this gap by developing integration indexes that quantify the extent of facility-level family planning and child immunization services integration as a varying and multifaceted outcome. Indexes like these may be used to analyze associations between the extent of integration and outcomes such as receipt of services, client satisfaction, and quality of care. The methodological process demonstrated in this study may be adapted by those aiming to better understand the extent of integration within facilities and over time.

## Methods

### Setting and data source

The data for this study were collected by the Measurement, Learning & Evaluation (MLE) project for the impact evaluation of the Nigerian Urban Reproductive Health Initiative (NURHI). NURHI, funded by the Bill & Melinda Gates Foundation, aimed to increase modern family planning use among the urban poor through a multi-pronged approach that included improving the quality of family planning services in high-volume urban health facilities. The intervention supported contraceptive supply chains and logistics, training for family planning counseling and provision, and facility level management systems. This study utilizes health facility (N = 400, 58% hospitals, 42% primary healthcare centers) and healthcare provider (N = 1479) baseline survey data that were collected in 2011 from Abuja (Nigeria’s capital), Benin City, Ibadan, Ilorin, Kaduna and Zaria [34]. NURHI selected these cities because they include both northern and southern regions of the country and each has a population of approximately, or more than, one million. The northern and southern regions of Nigeria differ in their cultural, economic, and religious characteristics; the north is poorer and predominantly Muslim while the south is more affluent and predominantly Christian.

### Study sample

Two categories of healthcare facilities are included in the sample: high-volume facilities (HVF) (n = 112) and preferred- provider facilities (PPF) (n = 288). HVF and PPF can be either public or private, and they can be either primary or secondary facilities. The sample includes all public facilities in the study cities. HVF, generally the top service delivery sites by client load, offered both antenatal care and immunization services; these facilities served more than 1000 antenatal clients per year. The NURHI program supported all HVF, and all of these facilities are included in the sample [34]. PPF were identified from a baseline household survey conducted by MLE that contained a representative sample of 16,144 women aged 15–49. Women were asked the name of each facility where they went for family planning, maternal health, and child health services. Using this information, MLE created a list of facilities that women reported by study cluster (primary sampling unit). The most commonly mentioned facility in each primary sampling unit was categorized as a PPF. If the PPF was already included in the sample as a public facility or an HVF, then the second most-commonly mentioned facility was included. If the second most-commonly stated facility was already in the sample, no additional facility was included. Including the PPFs along with the public facilities and HVFs ensures that the sample includes facilities that women in these urban areas actually visit.

### Survey instruments

This study utilizes instruments developed for the NURHI impact evaluation, which draws upon validated tools selected from the Quick Investigation of Quality [35]. Facility and provider surveys were conducted in each facility by trained interviewers hired by Data Research and Mapping Corporation; the MLE project provided technical assistance for training of interviewers. The surveys collected information on the readiness of facilities and providers to offer integrated services, usual or ‘normal’ family planning service provision practices in specific circumstances (e.g., usual or ‘normal’ practice within the facility if a woman has come for a child health service visit and is interested in receiving a hormonal method of contraception), gaps in commodities, equipment, training and resources, the extent of family planning integration into maternal, newborn and child health services, and other health facility characteristics. One facility audit was conducted per facility by asking questions of a manager or another administrator. In larger facilities, four providers were selected through simple random sampling to complete the provider survey; in the event that a provider declined another provider was randomly selected until four eligible providers consented to interview. In facilities with four or fewer providers, all were approached for interview. Providers eligible for inclusion were medically qualified to provide clinical services and assigned to provide direct family planning and/or maternal, newborn and child health services to clients at that facility; their responses are analyzed in this study.

### Statistical method

This study employs Principal Component Analysis (PCA) to create two family planning and child immunization services integration indexes: a Provider Integration Index and a Facility Integration Index. All analyses were conducted using Stata version 13.1 (Stata Corp, LP, College Station, Texas).

### Constructing and interpreting the indexes

#### Selection of variables for inclusion in the PCA

Drawing on Mayhew [30], we posit that numerous characteristics and processes interact within a health facility to result in varying degrees of integrated service delivery. While the Nigerian Ministry of Health does not provide a specific definition of integrated family planning and immunization services, their 2008 National Guidelines for the Integration of Reproductive Health and HIV Programmes offers this explanation:

Integration in the health sector has been defined by offering two or more services at the same facility during the same operating hour, with the provider of one service actively encouraging clients to consider using the other services during the same visit, in order to make those services more convenient and efficient. Integrated services should be offered at the same point but where that is not possible, strong referral systems are required to ensure that clients receive high quality service [36].

NURHI’s Strategy for Integrating Family Planning into Maternal, Newborn, Child Health and HIV/AIDS Services references this guidance [37]. This study also refers to this guidance to inform the attributes measured in the indexes. Additionally, we reviewed the integration literature to identify facility-level attributes that support service integration. Several critical attributes emerged, including (a) facility norms that support concurrent service provision (e.g., operational management standards and procedures that support the availability of both child immunization and family planning services at the same consultation or on the same day), and (b) provider capacity to offer multiple services (e.g., provider(s) has the skills and willingness to offer family planning information or services during a child immunization visit) [26, 30, 38–41].

Because this study was conceptualized after data collection, we leveraged the available data and selected eight indicators for inclusion in the indexes (Table 1). Table 2 describes these and other facility characteristics. A few of these indicators warrant additional explanation. The indicators used to develop the integration indexes focus primarily on family planning information and services that are provided during the child immunization visits. The indexes are thus most appropriate for use within that context, though they do also measure a range of referral scenarios. Improving outcomes through integration relies upon both high coverage and quality of integrated services. A substantial body of research links higher quality family planning services with increased contraceptive adoption, prevalence, and continuation; poor family planning service quality can hinder use [42]. Therefore, the level of quality provided and the absence of barriers that limit coverage and quality are essential indicators of effective integration [40, 43]. We analyzed quality of integrated family planning services by measuring the range and breadth of family planning topics that providers discuss with a client during child health service visits. Because the extent of integration can be influenced by provider bias [44–46], we include social norm-based service barriers by measuring the extent to which providers at a facility require partner consent prior to provision of a family planning method during an integrated visit. In Nigeria, partner consent is a tenacious barrier to contraceptive use that may be mitigated by training providers that standard service provision guidelines do not include a requirement for partner consent, providing supportive supervision on guideline implementation, and utilizing more comprehensive behavior change approaches with providers [47]. While numerous such barriers exist and could have been employed in the indexes this is the only variable in our dataset that captures such barriers to family planning specifically during immunization visits.

**Table 1:** Description of Principal Component Analysis input variables

Input variable description	Type	Survey source
What proportion of providers at facility offer both child immunization and family planning services?^a^	Continuous between 0 and 1	Provider
What proportion of providers at facility routinely offers family planning information during child immunization or child growth monitoring visits?^a^	Continuous between 0 and 1	Provider
What is the average count of family planning topics that providers at facility discuss with client during child health service visits?^b^	Ordinal between 0 and 7 FP topics include: (1) Identify reproductive goals (2) Provide information about different FP methods (3) Discuss the client's FP preferences (4) Help women select a suitable method (5) Educate women to use the selected method (6) Explain side effects (7) Explain specific medical reasons to return	Provider
What proportion of providers at facility do not request partner consent prior to woman’s receipt of family planning services during a child health service visit?^a^	Continuous between 0 and 1	Provider
Does the facility provide both child immunization and family planning services?	Binary (0 = no, 1 = yes)	Facility
What is the normal practice at this facility if client wants family planning information during a child health service visit?^c^	Ordinal between 0 and 7 Responses include: (0) Facility does not provide child health services (1) Facility does provide child health services but does not provide family planning services (2) Client is given no information or referral (3) Client is given referral to another facility (4) No appointment made, client told to return on a different day (5) Appointment made for different day (6) Client sometimes receives information on same day (7) Client always receives information on same day	Facility
What is the normal practice at this facility if client wants hormonal method of family planning during a child health service visit?^c^	Ordinal between 0 and 7 Responses include: (0) Facility does not provide child health services (1) Facility does provide child health services but does not provide family planning services (2) Client is given no information or referral (3) Client is given referral to another facility (4) No appointment made, client told to return on a different day (5) Appointment made for different day (6) Client sometimes receives method on same day (7) Client always receives method on same day	Facility
What is the score of operational days when both child immunization and family planning services are offered?^d^	Continuous between 0 and 1 Defined as: (Proportion of operational days that child immunization services are provided) multiplied by (Proportion of operational days that family planning services are provided)	Facility

*Child health* service visits include either immunization or growth monitoring visits, but not sick child visits. In variables referring to child health service visits, it was not possible to differentiate data pertaining only to immunization from data pertaining only to growth. Client exit interviews show that immunization visits comprise 95% of all child health service visits

^a^Proportion of providers was obtained from the provider survey by taking an average of provider responses to dichotomous survey questions (0 = no, 1 = yes). For example, if two providers responded that they did not routinely offer family planning information during an immunization or growth monitoring visit and two responded that they did then the facility would score a 0.5 on this item

^b^Facility score calculated by adding one point for affirmative responses to items 1–7

^c^Facility scores reflect the response, ranked from 0 (low) to 7 (high)

^d^The score for a facility that is open 7 days/week and offers immunization 1 day/week and family planning services 7 days/week would be calculated: (1/7) × (7/7) = 0.14

**Table 2:** Facility characteristics

Characteristic	Total (N = 400), n (%)
Facility type	
High volume	112 (28)
Preferred provider	288 (72)
Ownership	
Publicly owned	164 (41)
Privately owned	236 (59)
Level	
Primary	166 (42)
Secondary	234 (58)
Location	
Abuja	48 (12)
Benin	71 (18)
Ibadan	62 (15)
Ilorin	72 (18)
Kaduna	92 (23)
Zaria	55 (14)
Facilities that offer child immunization and/or child growth monitoring	339 (85)
Facilities that provide child immunization and family planning services	307 (77)

	Mean value (SD)
Normal practice if client is interested in family planning information during a child health service visit	5.82 (2.23)
Normal practice if client is interested in hormonal family planning during child health visit	4.63 (1.97)
Score of days where both child immunization and family planning are offered^a^	0.14 (0.38, 0.02–0.4)
Proportion providers at facility who offer child immunization and at least 1 modern family planning method	0.56 (0.40)
Proportion providers at facility who routinely offer family planning information during child immunization/growth monitoring	0.58 (0.39)
Average family planning topics that a provider at a facility discusses with client during child health service visit	1.67 (1.52)
Proportion providers at facility that do not request partner consent for family planning during child health service visit	0.50 (0.37)

See Table 1 for complete variable definitions

^a^Because of the preponderance of low scores, the median and interquartile range provide a clearer representation of the data for this item and are thus presented here

Several variables refer to child immunization, child growth monitoring, or child health service visits. Child health services visits include either immunization or growth monitoring visits, but not sick child visits. In variables referring to child health services, it was not possible to differentiate data pertaining only to child immunizations from data pertaining only to child growth monitoring. However, child immunization visits comprise the vast majority of all child health services visits. In the concurrent health facility client exit interview, 1714 people attended the facility for a child health service visit. Of these, only 90 (5%) report that child growth monitoring was the primary purpose of their visit while the remaining 95% reported immunization as the primary purpose of their visit. Facility-level variables are based on a summary of provider responses. Means were imputed for missing data.

#### PCA application

**Fig. 1:**
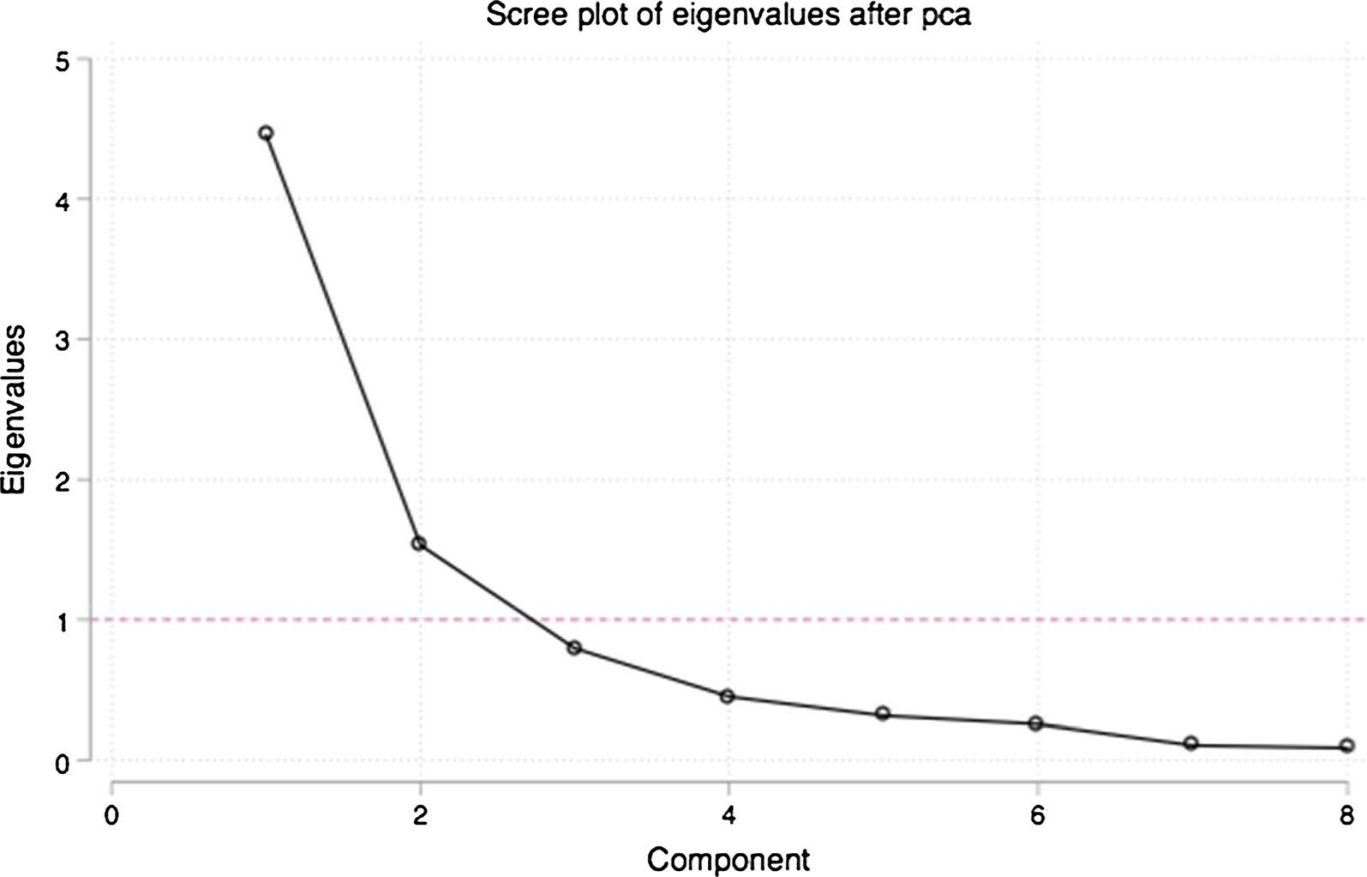
Scree plot of eigenvalues after principal component analysis

PCA was applied following the selection and transformation of variables. Input variables were standardized to a mean of zero and a standard deviation of one prior to the analysis to prevent variables with greater variance from dominating each component. The Kaiser–Meyer–Olkin (KMO) test of sampling adequacy was used to ascertain the suitability of the data for use in a PCA. Our KMO test yielded a score of 0.8, indicating sampling adequacy for each variable and the complete model. Based on evaluation of the eigenvalues (Table 3) and the scree plot (Fig. 1) we retained two components. The factor loading scores (see factor loadings column in Table 4), which show the correlation coefficient between each variable and component, were examined to determine which dimensions of integration are represented by the components. The scores confirmed the anticipated dimensions: provider integration and facility integration.

**Table 3:** Main Principal Component Analysis results from analysis of health facility data from six cities in Nigeria

Component	Eigenvalue	Proportion of explained variance	Proportion of cumulative explained variance
Comp1	4.456	0.557	0.557
Comp2	1.532	0.191	0.748
Comp3	0.795	0.099	0.848
Comp4	0.450	0.056	0.904
Comp5	0.315	0.039	0.943
Comp6	0.256	0.032	0.975
Comp7	0.107	0.013	0.989
Comp8	0.089	0.011	0.999

**Table 4:** Provider and Facility Integration Index Scores and  variable means by group

	Factor loading	Sum of factor loadings	Weight	Overall mean (SD)	Integration Index Score Classification Group Means			p-values		
					Low score (0–3.29)	Medium score (3.30–6.59)	High score (6.60–10)	Low–Med.		Med.–High
Provider integration variable description										
Proportion of providers at facility who offer child immunization and at least 1 modern family planning method	0.39	1.87	0.21	0.56 (0.4)	0.05	0.47	0.90	0.00	0.00	
Proportion of providers at facility who routinely offer family planning information during child immunization/growth monitoring	0.41	1.87	0.22	0.58 (0.39)	0.04	0.49	0.93	0.00	0.00	
Average number of family planning topics that a provider at a facility discusses with client during child health service visit	0.33	1.87	0.18	1.67 (1.52)	0.14	1.76	3.38	0.00	0.00	
What proportion of providers at facility do not request partner consent prior to woman’s receipt of family planning services during a child health service visit?	0.37	1.87	0.20	0.5 (0.37)	0.04	0.41	0.81	0.00	0.00	
Facility provides child immunization and family planning services	0.38	1.87	0.20	0.77 (0.42)	0.23	0.88	0.97	0.00	0.00	
Provider Integration Index score				5.42 (3.10)	0.75 (23% of facilities)	4.99 (32% of facilities)	8.23 (45% of facilities)	0.00	0.00	
Facility integration variable description										
Normal practice if client is interested in receiving family planning information during child health service visit	0.38	1.21	0.32	5.82 (2.23)	0.09	0.91	0.98	0.00	0.00	
Normal practice if client is interested in receiving hormonal family planning during child health service visit	0.41	1.21	0.34	4.63 (1.97)	0.05	0.80	0.96	0.00	0.00	
Score of days where both child immunization and family planning are offered	0.42	1.21	0.34	0.29 (0.35)	0.01	0.11	0.49	0.00	0.00	
Facility Integration Index Score				6.22 (2.72)	0.50 (14% of facilities)	6.00 (38% of facilities)	8.10 (48% of facilities)	0.00	0.00	

#### Creating the indexes

We constructed the Provider Integration Index and Facility Integration Index using weights calculated for each of the variables by dividing its factor loading by the sum of the factor loadings of all variables in that component (see weights column in Table 4). Next, we multiplied the variables included in each component by their associated weights and summed the values. Finally, we calculated the Provider Integration Index score and the Facility Integration Index score for each facility by multiplying these values by ten. The indexes thus range in value from zero to ten, with a higher score indicating a higher level of integration. Each facility was classified as having “low integration” (index score 0–3.29), “medium integration” (3.30–6.59) or “high integration” (6.60–10.00). These classifications were determined by dividing raw scores equally into tertiles along the score continuum of zero to ten. A sensitivity analysis was conducted to identify the effects of excluding from the sample those facilities that do not offer child immunization (n = 61); there were no statistically significant differences between the indexes that include all facilities versus those with the restricted set of facilities. We retained these facilities in the sample because one goal of the paper is to assess integration across the range of facilities and circumstances represented by our sample. Excluding these facilities would prevent us from knowing the full extent of integration across our sample. Additionally, one key benefit of developing these indexes is the ability to apply them to understand the effects of integration on health and service delivery outcomes. Having a score for facilities that do not offer child immunization allows future research to better identify correlations between level of integration (even very low level) and other outcomes.

#### Index coherence and robustness

Following Filmer and Pritchett [48], we assessed the internal coherence and robustness of the indexes. We examined internal coherence by comparing facility characteristics and index scores across low (0–3.29), medium (3.30–6.59), and high integration groups (6.60–10). We assessed robustness by examining how the classifications of facilities having high Integration Index scores changed when different sub-sets of variables were entered into the PCA. To assess the robustness of the Provider Integration Index, we ran 6 variations of the PCA. The first variation (“base case”) included all variables. Each subsequent model omitted one of the provider integration variables. Similarly, to assess the robustness of the Facility Integration Index, we ran 4 variations of the PCA. The first variation (the “base case”) included all variables. Each subsequent model omitted one of the facility integration variables. Looking only at the sites classified as “high integration” in the base case, we examine the impact on classification when we omit one variable at a time from the PCA. As an additional robustness check, we also created the indexes using Exploratory Factor Analysis to ascertain whether these indexes correlate with those created using PCA.

## Results

Eighty-five percent of facilities offer either child immunization or child growth monitoring services while 77% of facilities provide both family planning and child immunization services (Table 2). Private facilities are less likely than public facilities to provide both child immunization and family planning services; 93% of public facilities provide both child immunization and family planning services, while 66% of private facilities offer both services. Among the 93 facilities (81 = private, 12 = public) that do not offer both child immunization and family planning services, it is generally child immunization services that are not offered. Among the private facilities that do not offer both child immunization and family planning services, 89% do not offer child immunization services while 9% do not offer family planning services. Among the public facilities that do not offer both child immunization and family planning services, 50% do not offer child immunization services while 50% do not offer family planning services (data not shown). Among facilities that provide family planning services, 84% offer them either 5 or 7 days a week. Immunization provision is less frequent; 51% of the facilities that provide them do so once or twice per week while 26% provide them every day. On average, 58% of providers in each facility report offering family planning information during child health visits while 56% of providers in each facility have been trained to provide both child immunization and family planning services. Providers, on average, addressed fewer than two of the seven elements of family planning service provision during child health visits. Fifty percent of providers report requesting partner consent prior to providing family planning at a child health visit, though this varies by method.

**Fig. 2:**
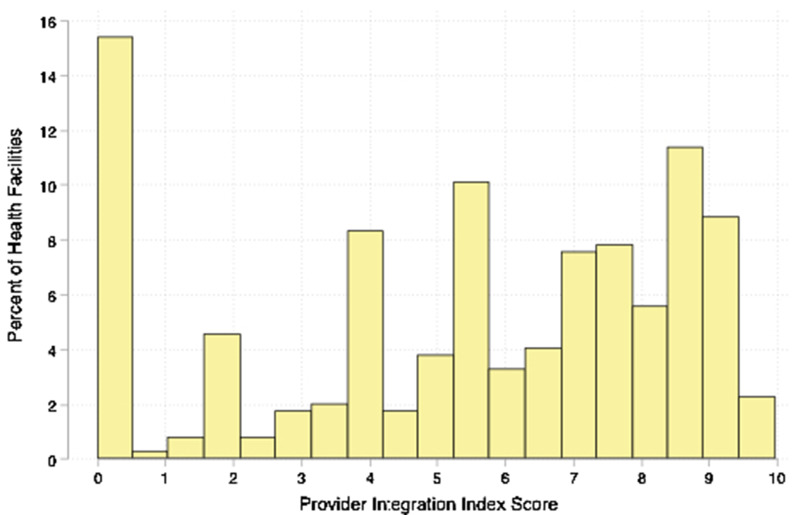
Distribution of Provider Integration Index scores across 400 health facilities in urban areas of Nigeria

**Fig. 3:**
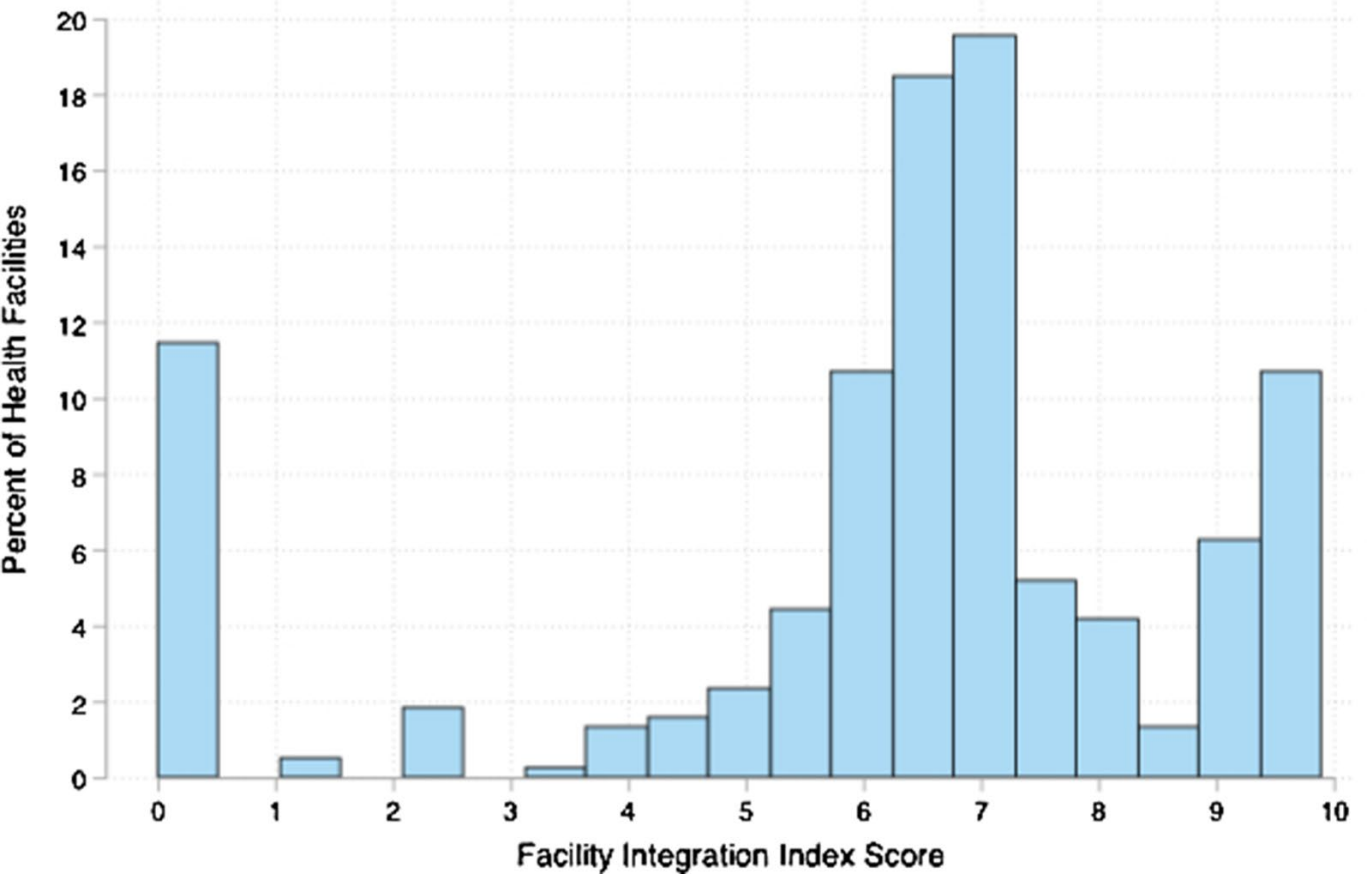
Distribution of Facility Integration Index scores across 400 health facilities in urban areas of Nigeria

Index scores vary across facilities (Figs. 2 and 3). Table 4 provides Provider and Facility Integration Index scores and their classifications. Please refer to the “Creating the Indexes” section for a description of how values in Table 4 were calculated and how these values link to the index scores. The mean Provider Integration Index score is 5.4 (standard deviation: 3.1, range: 0–10) and the mean Facility Integration Index score is 6.2 (standard deviation: 2.7, range: 0–9.9) (Table 4). Twenty-three percent of facilities are classified as having low Provider Integration Index scores, 32% are classified as having medium scores and 45% are classified as having high scores. Mean Provider Integration Index scores are 0.75, 4.99, and 8.23 for the low, medium, and high groups respectively. Fourteen percent of facilities are classified as having low Facility Integration Index scores, 38% have medium scores and 48% have high scores. The mean Facility Integration Index scores are 0.50, 6.00, and 8.10 for the low, medium, and high groups respectively. The mean value of each component within each classification group is presented within the Integration Index Score Classification Group Means columns. For example, on average, 4% of providers in facilities classified as having low integration index scores routinely offer family planning information during child immunization visits, 49% of providers in facilities with medium scores do, and 93% of providers in facilities with high integration index scores do. As another example, on average, providers in facilities classified as having low integration index scores discuss less than one (0.14) key family planning topic with clients during a child health services visit, while providers in facilities with medium integration index scores discuss fewer than two (1.76), and providers in facilities with high integration index scores discuss more than three (3.38) key topics.

### Internal coherence and robustness of the indexes

Both indexes demonstrate strong internal coherence. This is indicated by significant differences in facility characteristics and overall index scores across all groups for both indexes, meaning that the integration variables were significantly different between the low and medium score groups and the medium and high score groups (see p-values in Table 4). The Provider Integration Index is highly robust to the inclusion of different sub-sets of variables in the model. This is evidenced by the very similar classification results among facilities with high scores following the use of variable sub-sets. Table 5 shows the percent of facilities classified as “high integration” in the base case compared with the percent of facilities classified as “high integration” in the subsequent models, which each omit a variable. The table also shows the percent of facilities classified as “high integration” that shift to the “medium integration” in each of the subsequent models. For example, when the variable “proportion of providers who offer child immunization and at least one modern family planning method” is omitted from the PCA, 93% of the facilities that were classified as “high integration” in the base case remain in the “high integration” classification group while 7% shift to the “medium integration” group and 0% shift to the “low integration group”. Table 6 presents the same results for the Facility Integration Index. Unlike the Provider Integration Index, the Facility Integration Index shows considerable sensitivity to the different sub-sets of variables included in the PCA models. When the variables indicating normal practices at the facility are omitted, the Facility Integration Index score shifts towards the mean value of the score of operational days when both child immunization and family planning services are offered. Based on these assessments, we retained all base case variables in the model in order to reflect more characteristics of provider and facility integration [49]. Finally, the indexes created using EFA correlate strongly with the indexes created using PCA (Spearman rank correlation 0.99 for the Provider Indexes and 0.89 for the Facility Indexes), indicating that the results are robust to the use of either method.

**Table 5:** Provider Integration Index score classification differences in facilities classified as “high integration” in base case

Provider Integration Index score classification	Index score	Base case: all variables (%)	Omitted variable				
			Proportion providers who offer child immunization and at least 1 modern family planning method (%)	Proportion providers who routinely offer family planning information during child immunization and growth monitoring (%)	Average number of family planning topics that a provider discusses with client during child health service visit (%)	Proportion providers who do not request partner consent during child health service visit (%)	Facility provides both child immunization and family planning (%)
Low	0.00–3.29	0	0	0	0	0	0
Medium	3.30–6.59	0	7	5	0	5	24
High	6.60–10.00	100	93	95	100	95	76

**Table 6:** Facility Integration Index score classification differences in facilities classified as “high integration” in base case

Facility Integration Index score classification	Index score	Base case: all variables (%)	Omitted variable		
			Normal practice if client wants family planning information during child health service visit (%)	Normal practice if client wants to receive hormonal method of family planning child health service visit (%)	Score of days when both child immunization and family planning are offered (%)
Low	0.00–3.29	0	0	0	0
Medium	3.30–6.59	0	55	55	1
High	6.60–10.00	100	45	45	99

## Discussion

The purpose of this paper is to demonstrate the development of novel indexes that may be adapted and applied to measure integration along a continuum, and to apply these indexes to describe the extent of family planning and child immunization integration across a large sample of primary and secondary health facilities. The index scores allow for individual facility scoring and ranking; the components within the indexes enable even more nuanced analyses to inform policy and program strengthening. These integration indexes may be adapted and employed to enrich understanding of integration levels within and across health facilities, identify facilities for intervention, inform and monitor the effectiveness of interventions, and investigate the effect of integration on a range of service delivery and health outcomes. The NURHI survey instruments are publicly available and provide a valuable reference for the adaptation or development of indicators for future programs and research [50]. Some widely used surveys offer the opportunity to adapt this methodology to analyze integration of family planning into other services. For example, the Demographic and Health Survey (DHS) Program’s Service Provision Assessment (SPA) and the Performance Monitoring for Action (PMA) surveys include indicators that assess service availability, provider training and/or scope of practice, and client receipt of family planning information and services during other consultations, though not immunization services. Future revisions of these surveys may offer the opportunity to include questions related to immunization services, which could facilitate analyses about the integration of family planning and child immunization services. With these other survey tools, a similar non-binary index of integration can be created to assess family planning integration into other reproductive health services.

Our results suggest that facility norms and provider capacity to support integrated family planning and child immunization service delivery varies considerably across our sample. The identification of two distinct dimensions of integration, the heterogeneity of the scores and the substantial percentage of facilities within each integration index classification level, suggests that measuring integration as a binary variable does not reflect the true variation in integration within and across health facilities in urban areas of Nigeria. This is consistent with the findings of Mayhew [30] and Pfitzer and underscores the importance of employing ordinal or continuous measures of integration in research that examines the determinants and effects of integration [30, 46].

The development of the integration indexes required us to take a detailed look at provider and facility characteristics in each index. Several characteristics warrant particular attention. First, the majority of facilities with low Provider Integration Index scores (n = 94) do not provide both child immunization and family planning services, which is essential to integration. While we considered excluding these facilities from the sample, we opted to retain them because doing so enables description of the full spectrum of integration across our sample. Additionally, one benefit of these indexes is the ability to apply them to analyze the evolution of integration and its effects on a variety of outcomes. Having the complete range of scores allows future research to track integration in all of the facilities and identify correlations between level of integration (even very low level) and other outcomes. This is particularly important when considering that low levels of integration may impart some benefits, such as increased privacy, that may positively influence outcomes. Lastly, we retained these facilities in the sample because the Minimum Standards for Primary Healthcare in Nigeria stipulate that primary healthcare centers should provide both services, and both services form part of a standard package of primary health services provided by hospitals. Our results show that it is primarily private facilities that do not provide both family planning and immunization services. In Nigeria, routine immunization has been funded primarily by the government and Gavi, the Vaccine Alliance [51]. Private facilities may not qualify for either source of funding and may thus be less likely to offer immunization services. The private sector provides a substantial amount of health services in developing countries, including in urban areas of Nigeria [52]. Thus, it is important to understand its capacity to provide integrated services and consider whether and how private sector facilities might implement integration of family planning into other services. Secondly, the median score of the operational days when both child immunization and family planning services are offered (median = 0.14, interquartile range = 0.38 [0.02–0.40]) indicates that many facilities do not offer integrated services on a daily basis. Immunization services are provided on fewer days per week than family planning services, likely due to logistic issues such as reliance upon cold boxes that are delivered on specific days. Policies and programs supporting the integration of family planning and child immunization services could ensure that staffing and management procedures enable a full range of high-quality family planning services on immunization days.

Another factor we observed is the high percentage of providers requiring partner consent prior to providing a method of contraception. Our results show that 50% of providers report requiring partner consent during integrated visits; the percentage varies by contraceptive method selected. This substantial barrier to family planning service during integrated visits warrants additional investigation. Finally, it is important to consider the quality of family planning services when they are integrated into immunization services. Our results indicate that providers discuss a limited range of information with women during integrated visits. While provision of family planning information and services within the immunization setting may offer benefits, such as decreased need for repeat visits, it also presents challenges, such as potentially insufficient staffing, consultation time, and privacy to allow high quality information provision and discussion about women’s reproductive goals and needs [28]. This is an important consideration for the design of integrated service provision; integration approaches that establish easily accessible and efficient linkages between immunization and family planning services may prove more beneficial and be more likely to offer women a full range of methods than those which offer limited family planning information and services within the immunization setting. This is an important area for continuing research. Policies and programs promoting integration should ensure sufficient support to individual providers and facility systems to ensure that integration approaches enhance family planning service quality.

This study has several limitations. First, while the Nigerian Ministry of Health promotes the integration of family planning and child immunization services, it does not provide a precise definition of integration. We therefore relied upon Nigeria’s 2008 National Guidelines for the Integration of Reproductive Health and HIV Programmes (this, to our knowledge, is the most detailed and comprehensive definition of integration promoted by the Nigerian Ministry of Health), the NURHI integration strategy, and the broader integration literature to shape the attributes included in the indexes. This issue has challenged previous research; the lack of precise definitions of integration has long complicated its measurement [53]. Also, because this was a secondary analysis, we could not get precise information about some factors that are important to integration, such as service overlap. While we know the number of days per week immunization and family planning services are offered, we do not know *which* days of the week, so although family planning services are provided 5–7 days per week in the majority (approximately 85%) of facilities that provide family planning services, it is possible that we misrepresented the overlap in some instances (e.g., a facility that offers immunization on Monday and family planning on Monday, Wednesday and Friday would score the same as a facility that offers immunization on Tuesday and family planning on Monday, Wednesday and Friday). Future research could address this by collecting more specific information about the days and hours when these services are offered. Similarly, while we assume that providers are aware of normal practices in the facility, it is possible that they lack completely accurate knowledge. Either of these situations may lead to inaccuracies that would affect the index scores. Additionally, the provider and facility survey responses may be subject to social desirability bias. Future studies may address this issue by triangulating information between clients and providers. Our sampling strategy ensured inclusion of public facilities, which was NURHI’s focus, as well as private facilities that women actually attended. This provided an opportunity to collect relevant information about a variety of well-attended facilities across the six cities. However, the high number of private facilities precluded a census. Thus, our results are specific to our sample and are not generalizable to all urban facilities in Nigeria or other contexts. For example, because of NURHI’s focus on high-volume facilities, the sample includes 58% hospitals and thus over represents secondary facilities, which comprise 12% of total health facilities in Nigeria [54]. Last, this data was collected in 2011 and our results should be interpreted as a description of the extent of integration within these facilities at the time of data collection. Indeed, whether, how, and why the extent of integration changes within facilities over time is an important area for future research.

Notwithstanding these limitations, this research advances the conversation about how to measure integration by describing the development of more nuanced measures of integration that identify facility and provider attributes that support integrated service delivery. Such indexes are valuable because they allow for more detailed measurement of the determinants and effects of integration over time. This research also describes the nature and extent of family planning and child immunization services integration in six cities of Nigeria. This is important information in light of the Nigerian government’s goal to reduce MMR and IMR by increasing contraceptive prevalence, in part, by reaching more postpartum women through integration of family planning and child immunization services.

## Conclusion

Integration of family planning and immunization services is complex and challenging, and evidence about its effects on service delivery and health outcomes has been inconsistent. Previous research on the integration of family planning and child immunization has designated health facilities as integrated or not based on whether the facility received an intervention intended to increase integration. This study takes a novel approach by developing indexes that offer continuous measures of facility-level family planning and child immunization services integration and using the indexes to identify the extent of integration within a sample of 400 health facilities in six urban sites of Nigeria. Measuring the degree of integration is valuable as a means of monitoring health facility and system performance. Over time, such measurement will enable clearer understanding of the extent, patterns, and adoption of integration as well the effect of integration programs on on service delivery and health outcomes.

This research underscores the need for policies and programs seeking to promote or improve integration to start with a clear, context-specific definition and approach that recognizes the dual provider and facility dimensions. The definition should align with specific Ministry of Health and program objectives and strategies and should be captured in data collection initiatives from the outset. While the method outlined in this paper is most appropriate for programs that include a large number of facilities and have the necessary data collection and analytic expertise, smaller programs may incorporate some of the approaches highlighted in this paper. Such programs can articulate a precise and context-specific definition of integration and ensure that project implementation and routine monitoring and evaluation activities capture this definition with a variety of measures. Future research should analyze the determinants of integration and the effects of varying degrees of integration on service delivery and health outcomes. While this research focuses specifically on service delivery within health facilities, further research should examine how health systems pillars such as governance, human resources, logistics, financing, and information management systems influence integrated service delivery. A more robust evidence base is essential to the development of integration policies and programs that will increase contraceptive prevalence among postpartum women to promote healthy birth spacing and, ultimately, reduce maternal and infant morbidity and mortality globally.

## Data Availability

Data from this study and all documentation are available upon request through the MLE Dataverse website at: https://dataverse.unc.edu/dataverse/mle.

## Abbreviations

AIDS: Acquired immune deficiency syndrome
EFA: Exploratory factor analysis
FP: Family planning
HIV: Human immunodeficiency syndrome
HVF: High-volume facility
IMR: Infant mortality rate
KMO: Kaiser–Meyer–Olkin
MLE: Measurement, Learning & Evaluation
MMR: Maternal mortality ratio
MSPH: Minimum Standards for Primary Healthcare
NURHI: Nigerian Urban Reproductive Health Initiative
PCA: Principal Component Analysis
PPF: Preferred Provider Facility
SD: Standard deviation
WHO: World Health Organization

## References

[1] Trends in maternal mortality 2000 to 2017: estimates by WHO, UNICEF, UNFPA, World Bank Group and the United Nations Population Division. Geneva: World Health Organization; 2019. Licence: CCBY-NC-SA 3.0 IGO. https://www.who.int/reproductivehealth/publications/maternal-mortality-2000-2017/en/. Accessed 21 Jan 2021.

[2] National Population Commission (NPC) and ICF. Nigeria Demographic and Health Survey 2018. Abuja: NPC; 2019. https://www.dhsprogram.com/publications/publication-fr359-dhs-final-reports.cfm.

[3] Government of the Federal Republic of Nigeria. Integration of the SDGs into National Development Planning: a voluntary review. Abuja; 2020. https://sustainabledevelopment.un.org/content/documents/26210Main_Messages_Nigeria.pdf.

[4] Bhutta ZA, Das JK, Bahl R, Lawn JE, Salam RA, Paul VK (2014). Can available interventions end preventable deaths in mothers, newborn babies, and stillbirths, and at what cost?. Lancet (Lond, Engl).

[5] Family Planning 2020. Nigeria 2018 Core indicators 1–9 Country Fact Sheet 2018. http://www.familyplanning2020.org/sites/default/files/Nigeria%202018%201-9%20Handout.pdf. Accessed 28 Aug 2020.

[6] Nigeria Family Planning 2020 Commitment Update. 2017. http://www.familyplanning2020.org/sites/default/files/Nigeria_FP2020_Commitment_2017.pdf. http://www.familyplanning2020.org/nigeria. Accessed 21 Jan 2021. **(press release)**.

[7] Conde-Agudelo A, Rosas-Bermudez A, Kafury-Goeta AC (2006). Birth spacing and risk of adverse perinatal outcomes: a meta-analysis. JAMA.

[8] World Health Organization (2006). Report of a WHO technical consultation on birth spacing.

[9] Moore Z, Pfitzer A, Gubin R, Charurat E, Elliott L, Croft T (2015). Missed opportunities for family planning: an analysis of pregnancy risk and contraceptive method use among postpartum women in 21 low- and middle-income countries. Contraception.

[10] World Health Organization (2018). Working together: an integration resource guide for immunization services throughout the life course.

[11] World Health Organization. Global immunization coverage 1980 to 2019. 2019. https://www.who.int/data/gho/data/themes/immunization. Accessed 29 Mar 2020.

[12] World Health Organization, Nigeria: WHO and UNICEF estimates of immunization coverage: 2019 revision. Geneva: World Health Organisation; 2019. https://www.who.int/immunization/monitoring_surveillance/data/nga.pdf. Accessed 29 November 2020.

[13] Wallace AS, Ryman TK, Dietz V (2012). Experiences integrating delivery of maternal and child health services with childhood immunization programs: systematic review update. J Infect Dis.

[14] High-Impact Practices in Family Planning (HIP). Family planning and immunization integration: reaching postpartum women with family planning services. Washington, DC: USAID; 2013. http://www.fphighimpactpractices.org. Accessed 15 Jan 2021.

[15] Cooper CM, Fields R, Mazzeo CI, Taylor N, Pfitzer A, Momolu M (2015). Successful proof of concept of family planning and immunization integration in Liberia. Glob Health Sci Pract.

[16] Dulli L, Green M, Katz K. Increasing access to postpartum family planning services in Madagascar: assessing the feasibility and acceptability of immunization services as an entry point to family planning. Research Triangle Park, NC: Family Health International, Madagascar Ministry of Health and Family Planning, and Madagascar Institute of Public and Community Health. 2010. https://www.researchgate.net/publication/273692969_Increasing_access_to_postpartum_family_planning_services_in_Madagascar_assessing_the_feasibility_and_acceptability_of_immunization_services_as_an_entry_point_to_family_planning. Accessed 15 Oct 2018.

[17] Dulli LS, Eichleay M, Rademacher K, Sortijas S, Nsengiyumva T (2016). Meeting postpartum women's family planning needs through integrated family planning and immunization services: results of a cluster-randomized controlled trial in Rwanda. Glob Health Sci Pract.

[18] Huntington D, Aplogan A (1994). The integration of family planning and childhood immunization services in Togo. Stud Fam Plan.

[19] Cleland J, Shah IH, Daniele M (2015). Interventions to improve postpartum family planning in low- and middle-income countries: program implications and research priorities. Stud Fam Plann.

[20] Church K, Wringe A, Lewin S, Ploubidis GB, Fakudze P, Mayhew SH (2015). Exploring the feasibility of service integration in a low-income setting: a mixed methods investigation into different models of reproductive health and HIV care in Swaziland. PLoS ONE.

[21] Ahumuza SE, Rujumba J, Nkoyooyo A, Byaruhanga R, Wanyenze RK (2016). Challenges encountered in providing integrated HIV, antenatal and postnatal care services: a case study of Katakwi and Mubende districts in Uganda. Reprod Health.

[22] Nigerian Federal Ministry of Health. Nigeria family planning blueprint (scale up plan). Abuja. 2014. https://www.healthpolicyproject.com/ns/docs/CIP_Nigeria.pdf. Accessed 20 Jan 2021.

[23] Nigerian National Primary Healthcare Developement Agency. Minimum Standards for Primary Healthcare in Nigeria. Abuja. 2012. https://www.hfr.health.gov.ng. Accessed 20 Jan 2021.

[24] Kuhlmann AS, Gavin L, Galavotti C (2010). The integration of family planning with other health services: a literature review. Int Perspect Sex Reprod Health.

[25] Speizer IS, Fotso JC, Okigbo C, Faye CM, Seck C (2013). Influence of integrated services on postpartum family planning use: a cross-sectional survey from urban Senegal. BMC Public Health.

[26] Vance G, Janowitz B, Chen M, Boyer B, Kasonde P, Asare G (2014). Integrating family planning messages into immunization services: a cluster-randomized trial in Ghana and Zambia. Health Policy Plan.

[27] Blazer C, Prata N (2016). Postpartum family planning: current evidence on successful interventions. Open Access J Contracept.

[28] Nelson AR, Cooper CM, Kamara S, Taylor ND, Zikeh T, Kanneh-Kesselly C (2019). Operationalizing integrated immunization and family planning services in rural liberia: lessons learned from evaluating service quality and utilization. Glob Health Sci Pract.

[29] Mounier-Jack S, Mayhew SH, Mays N (2017). Integrated care: learning between high-income, and low- and middle-income country health systems. Health Policy Plan.

[30] Mayhew SH, Ploubidis GB, Sloggett A, Church K, Obure CD, Birdthistle I (2016). Innovation in evaluating the impact of integrated service-delivery: the integra indexes of HIV and reproductive health integration. PLoS ONE.

[31] Mackenzie D, Pfitzer A, Maly C, Waka C, Singh G, Sanyal A (2018). Postpartum family planning integration with maternal, newborn and child health services: a cross-sectional analysis of client flow patterns in India and Kenya. BMJ Open..

[32] Hoang T, Goetz MB, Yano EM, Rossman B, Anaya HD, Knapp H (2009). The impact of integrated HIV care on patient health outcomes. Med Care.

[33] Mattocks KM, Kroll-Desrosiers A, Kinney R, Singer S (2019). Understanding maternity care coordination for women veterans using an integrated care model approach. J Gen Intern Med.

[34] Measurement, Learning & Evaluation (MLE) Project; National Population Council (NPC). 2010–2011 Nigeria baseline survey for the Urban Reproductive Health Initiative. Chapel Hill (NC): MLE Project; 2011. https://www.nurhitoolkit.org/document-library/nurhi-baseline-facility-report#.YAiqZi9h1R4.

[35] MEASURE Evaluation. Quick investigation of quality (QIQ): a user’s guide for monitoring quality of care in family planning. Chapel Hill, North Carolina: MEASURE Evaluation, University of North Carolina; 2016.

[36] Nigeria Federal Ministry of Health. National Guidelines for the Integration of Reproductive Health and HIV programmes in Nigeria. Abuja. 2008. https://www.childrenandaids.org/sites/default/files/2017-05/Nigeria-Integrated-National-Guildlines-For-HIV-Prevention-treatment-and-care-2014.pdf. Accessed 21 Jan 2021.

[37] NURHI. Strategy for integrating family planning into maternal, newborn, child health and HIV services, Abuja, 2009. https://www.nurhitoolkit.org/document-library/fp-integration-strategy#.X9fddC9h1R4. Accessed 25 Sept 2019.

[38] Dudley L, Garner P. Strategies for integrating primary health services in low‐ and middle‐income countries at the point of delivery. Cochrane Database Syst Rev. 2011(7). Art. No.: CD003318. 10.1002/14651858.CD003318.pub3. 10.1002/14651858.CD003318.pub3 PMC6703668 21735392

[39] Watt N, Sigfrid L, Legido-Quigley H, Hogarth S, Maimaris W, Otero-Garcia L (2017). Health systems facilitators and barriers to the integration of HIV and chronic disease services: a systematic review. Health Policy Plan.

[40] Kerber KJ, de Graft-Johnson JE, Bhutta ZA, Okong P, Starrs A, Lawn JE (2007). Continuum of care for maternal, newborn, and child health: from slogan to service delivery. Lancet (Lond, Engl).

[41] Mayhew SH, Sweeney S, Warren CE, Collumbien M, Ndwiga C, Mutemwa R (2017). Numbers, systems, people: how interactions influence integration. Insights from case studies of HIV and reproductive health services delivery in Kenya. Health Policy Plan.

[42] RamaRao S, Lacuesta M, Costello M, Pangolibay B, Jones H (2003). The link between quality of care and contraceptive use. Int Fam Plan Perspect.

[43] Curry N, Ham C (2010). Clinical and service integration: the route to improved outcomes.

[44] de Jongh TE, Gurol-Urganci I, Allen E, Jiayue Zhu N, Atun R (2016). Barriers and enablers to integrating maternal and child health services to antenatal care in low and middle income countries. BJOG Int J Obstet Gynaecol.

[45] Dynes MM, Bernstein E, Morof D, Kelly L, Ruiz A, Mongo W (2018). Client and provider factors associated with integration of family planning services among maternal and reproductive health clients in Kigoma Region, Tanzania: a cross-sectional study, Apri–July 2016. Reprod Health.

[46] Pfitzer A, Maly C, Tappis H, Kabue M, Mackenzie D, Healy S (2019). Characteristics of successful integrated family planning and maternal and child health services: findings from a mixed-method, descriptive evaluation. F1000Res.

[47] Solo J, Festin M (2019). Provider bias in family planning services: a review of its meaning and manifestations. Glob Health Sci Pract.

[48] Filmer D, Pritchett LH (2001). Estimating wealth effects without expenditure data—or tears: an application to educational enrollments in states of India. Demography.

[49] Vyas S, Kumaranayake L (2006). Constructing socio-economic status indices: how to use principal components analysis. Health Policy Plan.

[50] Measurement, Learning, and Evaluation Project, NURHI, & Data Research and Mapping Consult. Report of the baseline facility survey for the Nigerian Urban Reproductive Health Initiative (NURHI). 2011. http://www.nurhitoolkit.org/document-library/nurhi-baseline-facility-report#.WYx9LlKZN8c. Accessed 10 Dec 2020.

[51] Olumide F, Chidiabere O, Akinyede O, Botchway P, Soyemi K (2017). Vaccine financing in Nigeria: are we making progress towards self-financing/sustenance?. Pan Afr Med J.

[52] Levy JK, Curtis S, Zimmer C, Speizer IS (2014). Assessing gaps and poverty-related inequalities in the public and private sector family planning supply environment of urban Nigeria. J Urban Health.

[53] Atun R, de Jongh T, Secci F, Ohiri K, Adeyi O (2010). A systematic review of the evidence on integration of targeted health interventions into health systems. Health Policy Plan.

[54] Makinde OA, Sule A, Ayankogbe O, Boone D (2018). Distribution of health facilities in Nigeria: implications and options for universal health coverage. Int J Health Plan Manag.

